# Therapeutic role of neural stem cells in neurological diseases

**DOI:** 10.3389/fbioe.2024.1329712

**Published:** 2024-03-07

**Authors:** Ling Yang, Si-Cheng Liu, Yi-Yi Liu, Fu-Qi Zhu, Mei-Juan Xiong, Dong-Xia Hu, Wen-Jun Zhang

**Affiliations:** ^1^ Department of Rehabilitation Medicine, The Second Affiliated Hospital, Nanchang University, Nanchang, Jiangxi, China; ^2^ Department of Physical Examination, The Second Affiliated Hospital, Nanchang University, Nanchang, Jiangxi, China; ^3^ The Second Affiliated Hospital, Nanchang University, Nanchang, Jiangxi, China

**Keywords:** NSCs, neurological diseases, treatment, transplantation, challenge

## Abstract

The failure of endogenous repair is the main feature of neurological diseases that cannot recover the damaged tissue and the resulting dysfunction. Currently, the range of treatment options for neurological diseases is limited, and the approved drugs are used to treat neurological diseases, but the therapeutic effect is still not ideal. In recent years, different studies have revealed that neural stem cells (NSCs) have made exciting achievements in the treatment of neurological diseases. NSCs have the potential of self-renewal and differentiation, which shows great foreground as the replacement therapy of endogenous cells in neurological diseases, which broadens a new way of cell therapy. The biological functions of NSCs in the repair of nerve injury include neuroprotection, promoting axonal regeneration and remyelination, secretion of neurotrophic factors, immune regulation, and improve the inflammatory microenvironment of nerve injury. All these reveal that NSCs play an important role in improving the progression of neurological diseases. Therefore, it is of great significance to better understand the functional role of NSCs in the treatment of neurological diseases. In view of this, we comprehensively discussed the application and value of NSCs in neurological diseases as well as the existing problems and challenges.

## 1 Introduction

Neurological diseases, including Alzheimer’s disease (AD), Parkinson’s disease (PD), amyotrophic lateral sclerosis (ALS) and Huntington’s disease (HD), are highly crippled and fatal diseases that affect millions of people around the world (Heemels, 2016). The pathological mechanism of neurological diseases is mainly due to the progressive loss of neuronal structure or function, resulting in varying degrees of paralysis, cognitive, sensory and motor dysfunction, and even permanent disability (Heemels, 2016). Importantly, the ability of tissue repair and regeneration of neurological diseases is limited, and there is no radical cure that can stop or reverse their development. Clinical use of drugs to alleviate or control the symptoms of patients, but the effect of drug treatment is limited, still cannot achieve the desired effect. Importantly, drugs cannot repair damaged nerves and reconstruct neural networks. In addition, because the drug development process is complex and expensive, the clinical treatment of these diseases is limited. Therefore, there is an urgent need to explore effective treatment methods.

In recent years, with the continuous exploration of the repair of nerve injury, cell transplantation/cell therapy has entered people’s field of vision, has been widely used in neurological diseases, and broadened new treatment strategies. The therapeutic value of cell therapy in neurological diseases is to imitate the normal process of cell repair and development in the nervous system, so as to eliminate the causes of the disease, improve dysfunction and repair damaged tissue (Alessandrini et al., 2019; Andrzejewska et al., 2021). At present, researchers have achieved exciting results by transplanting different types of bioactive cells into nerve injury sites, such as spinal cord injury and sciatic nerve injury (Goulart et al., 2016; Ramalho et al., 2021). Neural stem cells are a kind of neuroblasts, which have the potential of self-renewal and differentiation, and can differentiate into neurons and glial cells (Merkle and Alvarez-Buylla, 2006). NSCs play an important role in the development of central nervous system. The functions of NSCs in the treatment of neurological diseases include promoting nerve regeneration, cell differentiation, secreting neurotrophic factors, neuroprotection and immune regulation, and producing pharmacological properties of repairing tissue damage and improving function (Dong and Yi, 2010; de Freria et al., 2021; Zhang et al., 2021). Importantly, NSCs therapy can replace endogenous deletion cells to reconstruct the function of neural network (Bellenchi et al., 2013). NSCs transplantation can replace injured or missing neurons by differentiating into neurons at the site of nerve injury, producing protective neurons and maintaining the number of neurons, which provides another attractive treatment strategy for the treatment of neurological diseases. Currently, different studies have confirmed the therapeutic effect and application value of NSCs in neurological diseases, and great progress has been made (Nizzardo et al., 2014; Ager et al., 2015; Lee et al., 2015; Mendes-Pinheiro et al., 2018). It reveals the fact that NSCs can be used in the treatment of neurological diseases. Therefore, here, we deeply discuss the application value of NSCs in the treatment of neurological diseases and some problems that need to be solved at present.

## 2 NSCs and their biological characteristics in nerve regeneration and repair

The journey of neural development begins with neuroepithelial cells, a kind of pluripotent neural stem cells, pseudo-layered cells arranged in the neural tube chamber. Neuroepithelial cells begin to divide symmetrically and maintain stem cell characteristics (Haubensak et al., 2004). After the formation of neural tube, neuroepithelial cells are transformed into radial glial cells and produce offspring and intermediate neural progenitor cells by asymmetric division, which differentiate into neurons and induce neuronal migration (Noctor et al., 2004; Gleason et al., 2008). With the development of embryo, radial glial cells can differentiate into oligodendrocytes and astrocytes (Okamoto et al., 2013). Near birth, radial glial cells change their characteristics to produce neural stem cells, which are libraries of neurogenesis and embryogenesis in adult life (Alvarez-Buylla et al., 2001; Fujita, 2003).

NSCs from adult mammals have unique characteristics, such as differentiation ability and self-renewal. They exist in specific gaps, namely, the subventricular zone (SVZ), located in the ependymal cell layer, separating the ependymal and subventricular zones, and the subgranular area (SGZ)-the dentate gyrus of the hippocampus (Gonzalez et al., 2016; Grochowski et al., 2018). The limited regeneration ability of the nervous system may be due to the limited number of NSCs in discrete locations, or surrounded by microenvironments that do not support neuronal differentiation, resulting in endogenous repair failure (Gonzalez et al., 2016). As a kind of self-renewing pluripotent cells, NSCs can produce various types of cells needed to establish the central nervous system (Kageyama et al., 2006). NSCs produce the plasticity of new neurons and glial cells through multiple processes and play an important role in embryonic development and adult neurogenesis. In recent years, NSCs transplantation has gained extensive recognition and application value in the application of tissue injury repair and nerve regeneration. Transplantation of NSCs into the injured spinal cord can increase the possibility of regeneration by replacing lost neurons or oligodendrocytes (Ishii et al., 2006; Xue et al., 2020).

In addition to the differentiation and self-renewal characteristics of NSCs, NSCs play a variety of functional characteristics in neural injury repair and regeneration, including immune regulation (the interaction between immune cells and NSCs/neural progenitor cells and their offspring seems to determine the effectiveness of endogenous regeneration response. It also determines the fate and functional integration of transplanted NSCs (Kokaia et al., 2012), secretion of neurotrophic factors (such as GDNF and BDNF) (Fu et al., 2011), anti-inflammatory regulation (Park et al., 2021), angiogenesis (Moshayedi and Carmichael, 2013), axon regeneration and myelin formation (Moshayedi and Carmichael, 2013; Chung et al., 2022) and inhibition of glial scar formation (Ishii et al., 2006) (Table 1). Moreover, in peripheral neurological diseases, implanted NSCs can differentiate into Schwann cells (SCs) and maintain a microenvironment rich in growth factors, thus promoting nerve regeneration (Lee et al., 2017). Studies have shown that IL12p80 can induce mouse NSCs to differentiate into SCs through STAT3 phosphorylation and promote the functional recovery of regenerated nerves after sciatic nerve injury (Lee et al., 2017). In view of these characteristics, the application value of NSCs in neurological diseases has a broad prospect.

**TABLE 1:** Functional characteristics of NSCs in nerve injury regeneration and tissue repair.

1. NSCs have the potential of self-renewal and differentiation, which can produce various types of cells needed to establish the central nervous system and maintain the development of the central nervous system
2. NSCs can produce new plasticity of neurons and glial cells
3. They have immunomodulatory and anti-inflammatory effects, which can improve the local microenvironment of nerve injury and is beneficial to the integration of cell function
4. They have the function of secreting a variety of neurotrophic factors (such as BDNF, NGF and GDNF) and provides a good groundwork for nerve regeneration
5. NSCs have the function of promoting vascular growth
6. Promote new axon regeneration and re-myelination, and produce neuroprotective effect
7. The function of inhibiting the formation of glial scar is beneficial for newborn axons to bridge long-distance axons through the scar area and form “bridging"
8. Differentiate into SCs, maintain a microenvironment rich in growth factors, and promote the repair of nerve injury
9. NSCs transplantation can replace/lose host neurons by differentiating into neuronal subtypes

## 3 The source of NSCs

NSCs mainly come from endogenous NSCs (human embryonic stem cells and human fetal brain NSCs/progenitor cells), induced pluripotent stem cells (iPSCs) and direct reprogramming (Zhang and Jiao, 2015; Grochowski et al., 2018). These provide a strong basis for the origin of NSCs and their development in the field of tissue repair and regeneration. During embryonic development, there are two key proliferative regions: the ventricular zone (VZ) and the subventricular zone (SVZ). NSCs are located in the VZ of the neural tube and produce various types of cells needed for the construction of the central nervous system (Pevny and Rao, 2003; Kageyama et al., 2006). NSCs in VZ divide symmetrically and asymmetrically to preserve stem cell banks, which then migrate to SVZ and then perform the ability to proliferate or differentiate (Guillemot, 2005). SVZ may be a special region. SVZ in the adult brain is the site of significant proliferation of neuronal cells in the early stage of development, which contains static NSCs. These endogenous resting NSCs are activated and participate in the repair process after central nervous system injury (Ito and Suda, 2014). Another major area of NSCs in the adult brain is the subgranular region of the dentate gyrus (Kriegstein and Alvarez-Buylla, 2009; Feliciano et al., 2015). NSCs can be obtained from adult subventricular zone or subgranular zone. NSCs can continuously produce and differentiate into functional neurons in the course of life, which have important functions on human body, including learning, memory and cognition (Gage and Temple, 2013).

In order to isolate, culture and obtain high yield NSCs from the subventricular zone or subgranular zone, the NSCs isolated from the subventricular zone or subgranular zone were cultured into neurospheres or monolayers, and then identified by Nestin, Sox2 or Msi1 labeling (Zhang and Jiao, 2015). The spheroids of NSCs can be well cultured and better characterized by the addition of epidermal growth factor, fibroblast growth factor 2 and B27 to the culture system *in vitro* (Gage and Temple, 2013; Zhang et al., 2021). After the NSCs isolated from the lateral ventricle of adult mice were cultured in a specific medium with growth factors, the number of neurospheres increased significantly (Stipcevic et al., 2013). Intracerebroventricular injection of 300,120 ng/d di-rhamnolipid could increase the number of nerve balls in the anterior lateral ventricle of adult mice (Stipcevic et al., 2013). These studies have revealed that NSCs can be obtained from endogenous, expanded or established into stable cell lines *in vitro*, and then transplanted to the injured site for regeneration treatment.

Direct cell reprogramming is based on the dominant role of cell pedigree transcription factors in transforming adult somatic cells into different cell types. This technique represents a promising approach in the field of regenerative medicine, with the potential to produce cell sources for cell replacement therapy (Graf and Enver, 2009; Chambers and Studer, 2011). Direct cell reprogramming can use the least set of cell line-specific transcription factors to directly transform fibroblasts into NSCs and differentiate into functional neurons and oligodendrocytes (Ring et al., 2012; Caiazzo et al., 2015). This method is fast and simple, and it takes only one step to generate the required NSCs. Adult primary fibroblasts can effectively directly transform into neural progenitor cells by timely limiting the expression of Oct4, Sox2, Klf4 and c-Myc, and show the morphological and molecular characteristics of NSCs/neural progenitor cells, such as Nestin and Sox2 (Meyer et al., 2015). Reprogrammed NSCs/neural progenitor cells can differentiate into neurons and astrocytes [Meyer S]. Studies have shown that mouse and human fibroblasts can be directly reprogrammed into NSCs without intermediate pluripotency (Kim et al., 2017). Human umbilical cord blood cells were directly reprogrammed into NSCs using pedigree-specific transcription factors SOX2 and HMGA2 with high efficiency and short turnaround time. The reprogrammed NSCs can be cloned and expanded and have the multipotential to produce neurons and glial cells (Zhang et al., 2022). Direct reprogrammed NSCs do not go through the multi-functional phase and are a safe and timely resource. This is an important progress in the field of cell transplantation therapy, and it also means that direct reprogramming of somatic cells provides a strong guarantee for NSCs for the treatment of nervous system diseases.

Induced pluripotent stem cells (iPSCs) have made an inestimable contribution to the field of regenerative medicine, bypassing ethical issues in the use of human embryos (Kim et al., 2017; Aboul-Soud et al., 2021). iPSCs have the plasticity of generating, repairing and altering nervous system function. Combined with reprogramming techniques, neural stem cells derived from human somatic cells and their offspring can more accurately simulate neurological diseases (Gage and Temple, 2013; Aboul-Soud et al., 2021). iPSCs can create specific populations of neurons, especially for neurodevelopment and the treatment of neurological diseases. iPSCs were induced from mouse embryonic or adult fibroblasts. These cells showed the morphological and growth characteristics of embryonic stem cells and expressed the characteristics of embryonic stem cell marker genes (Takahashi and Yamanaka, 2006). iPSCs were subcutaneously transplanted into nude mice to produce tumors containing various tissues of all three reproductive layers (Takahashi and Yamanaka, 2006). After further injection of these cells into blastocysts, the induction of pluripotent stem cells is helpful to the embryonic development of mice (Takahashi and Yamanaka, 2006). Studies have shown that iPSCs can produce powerful and cost-effective NSCs/early neural progenitor cells, which can differentiate into vesicular glutamate transporter 1 (VGLUT1) positive neurons (DAiuto et al., 2014). Human iPSCs-induced NSCs showed stable neural phenotypes. When these NSCs were transplanted into the model of ischemic stroke, it was found that these cells could survive and migrate to the ischemic area, and could differentiate into mature nerve cells, which was beneficial to the functional recovery (Liu, 2013). Moreover, the iPSCs derived NSCs/neural progenitor cells have low immunogenicity and can increase the survival rate after cell transplantation (Itakura et al., 2017). One of the advantages of iPSCs is that it removes ethical issues and restrictions on the use of embryonic stem cells. However, there are also some problems in iPSCs, such as genetic/epigenetic abnormalities and tumorigenesis caused by artificial induction, the progression of pluripotent state and subsequent complete differentiation to the desired lineage are still obstacles to the clinical translation of iPSCs technology, which must be solved before clinical use.

## 4 NSCs and neurological diseases

Neurological diseases include PD, AD, HD and ALS. These diseases are characterized by the loss of specific neuronal subsets, resulting in gradual loss and paralysis of sensory, cognitive and motor neurons (Fayazi et al., 2021; Liao et al., 2022). This kind of disease affects tens of millions of people around the world. with the aging of the world population, the incidence of this kind of disease continues to rise, bringing huge mental, physical and economic burden to patients (Marsh and Blurton-Jones, 2017). Despite the increasing understanding of the pathogenesis of these diseases, there is still a lack of effective and feasible treatments. Some drugs are used clinically to slow down the progression of these diseases, but the long-term use of drugs brings dependence, side effects and short-term benefits, so it is impossible to replace and recover the lost neurons and reconstruct the neural functional network. Therefore, with the continuous research and exploration of researchers, the strategy of cell therapy is introduced. People transplant functional active cells into the site of nerve injury to play a therapeutic role. Cell therapy treats neurological diseases by generating specific neurons to maintain and replace lost host neurons, establishing auxiliary connection neural networks around the affected areas, and creating a new environment to support the survival of host neurons (Sugaya and Vaidya, 2018). As a special kind of stem cells, NSCs have been widely studied in the field of nerve regeneration, which play a useful role through different mechanisms, such as the production of neurotrophic factors, reduction of neuroinflammation, neuroprotection and immune regulation (Grochowski et al., 2018; De Gioia et al., 2020; Zhang et al., 2020). In addition, NSCs must properly migrate to the injured nervous system and integrate into the host neuron circuit to reconstruct the neural network lost in the disease and enhance neural plasticity (Marsh and Blurton-Jones, 2017; De Gioia et al., 2020). Although the application of NSCs in neurological diseases is in the initial stage of exploration, it is a feasible new strategy for treatment (Table 2).

**TABLE 2:** preclinical study of neural stem cells in neurodegenerative diseases.

Cell source	Disease model type	Transplantation method	Treatment result	Refs
hVM1 clone 32 cells	Mouse PD model	Cells were injected into the left back of mice	NSCs were transplanted into PD mice to express dopamine-centered markers, the number of astrocytes and microglia increased significantly, and the weight of non-phosphorylated neurofilaments in motor cortex increased, which improved hyperactivity and gait changes in mice	Nelke et al. (2022)
NSCs conditioned medium	Rat PD model	Injection of conditioned medium of NSCs into striatum and substantia nigra	Transplantation of conditioned medium of NSCs improves motor and non-motor dysfunction in PD rats and saves the loss of dopaminergic neurons	Ni et al. (2022)
Neurospheres derived from embryonic stem cells	Mouse AD model	Transplanted to the frontal lobe joint cortex and barrel lobe cortex of mice	The transplantation of neurospheres into the prefrontal and parietal cortex significantly alleviated cholinergic defects and short-term memory impairment in NBM-damaged mice	Wang et al. (2006)
NSCs derived from embryonic stem cells	Rat AD model	Transplanted to rat striatum	After transplantation of neural progenitor cells and/or NSCs, the memory function of rats was significantly improved. Most (about 70%) of neural progenitor cells or NSCs retained neuronal phenotype after transplantation, of which about 40% had cholinergic phenotype and no tumor formation	Moghadam et al. (2009)
NSCs from hippocampus	Rat AD model	Transplanted into the basal forebrain of rats	The transplanted NSCs can differentiate into cholinergic neurons. NSCs transplantation can significantly increase the number of cholinergic neurons in medial septal nucleus and vertical branch of diagonal branch in rats	Xuan et al. (2009)
Human NSCs	Mouse AD model	Repeated intranasal injection of NSCs into the brain of mice	hNSCs transplanted into nasal cavity survived and showed extensive migration and high neuronal differentiation, but glial differentiation was relatively limited. Intranasal transplantation of hNSCs can partially differentiate into cholinergic neurons, thus saving cholinergic dysfunction in APP/PS1 mice	Lu et al. (2021)
Induction of neural progenitor cells derived from pluripotent cells	Rat ALS model	Intramedullary injection of cells	Cell transplantation significantly retained MNS, slowed the progression of the disease, and prolonged the survival time of all treated animals. The transplanted cells can normalize the expression of host genes (versican, has-1, tenascin-R, NGF, IGF-1, BDNF, bax, bcl2 and Casp-3) and protect the neural network around the preserved MN.	Forostyak et al. (2020)
NSCs derived from human embryonic spinal cord	Rat ALS model	HNSCs lumbar vertebra injection	The transplantation of hNSCs into the lumbar spinal cord of SOD1G93A rats can protect the α motoneurons near the transplanted cells and provide a transient functional improvement, but it has no protective effect on the α motoneurons far from the lumbar segment of the graft	Hefferan et al. (2012)
Human NSCs	Rat ALS model	Cells were transplanted into the anterior horn of lumbar spinal cord in rats	hNSCs was widely integrated in the spinal cord, differentiated into neurophenotype and migrated from the rostral end to the caudal end, which delayed the loss of body weight and the deterioration of motor ability in SOD rats	Zalfa et al. (2019)
NSCs derived from human embryonic stem cells	Mouse HD model	Transplanted into the striatum of mice	NSCs transplantation improved motor disorders, saved synaptic changes, and connected through the nerve endings of mouse cells	Reidling et al. (2018)
Cloned and conditionally immortalized neural stem cell line	Rat HD model	Transplanted into rat striatum and cortex	The transplanted cells formed MSN and GABA neurons expressing BDNF in the striatum, while the cells transplanted in the cortex formed Tbr1 positive neurons. The stable implantation and connection between the transplanted cells and the host brain tissue also reduced the formation of glial scar and inflammation, and increased endogenous neurogenesis and angiogenesis	Yoon et al. (2020)
Collection of NSCs from fetal rats in pregnant Fischer rats	Rat tibial nerve injury model	Transplantation to tibial nerve injury	Embryonic NSCs can be transplanted into peripheral nerve to differentiate into neurons and form functional neuromuscular connections with denervated muscles to prevent muscle atrophy	Gu et al. (2010)
NSCs isolated from the brain of newborn mice	Rat model of sciatic nerve injury	Transplanted into the injured sciatic nerve	NSCs combined with estradiol significantly restored motor nerve conduction velocity, voltage amplitude and motor tolerance in rats. The amount of blood vessels and blood perfusion in the nerve increased, and NSCs was recruited into the new blood vessels	Sekiguchi et al. (2013)
Rat-derived NSCs	Rat model of recurrent laryngeal nerve transection	Transplantation of laminin-chitosan-PLGA nerve conduit containing Schwann cells and NSCs into rat recurrent laryngeal nerve defect	After transplantation of laminin-chitosan-PLGA nerve conduit containing SCs and NSCs, the diameter and area of regenerated myelin sheath of recurrent laryngeal nerve and the secretion of brain-derived neurotrophic factor and glial cell line-derived neurotrophic factor in laryngeal muscle or regenerated nerve tissue were significantly increased, which promoted the regeneration of nerve injury	Li et al. (2020b)
NSCs derived from brain of F1B-Tag transgenic mice	A model of sciatic nerve injury in mice	Combined transplantation of NSCs, neural conduit and IL12p80 to the injured site of sciatic nerve	The combined transplantation of NSCs with neural conduit and IL12p80 can promote motor recovery and increase the medial diameter of the regenerated nerve by 4.5 times. IL12p80 can induce mouse NSCs to differentiate into SCs by STAT3 phosphorylation, and promote the functional recovery and diameter increase of regenerated nerve after sciatic nerve injury	Lee et al. (2017)

### 4.1 NSCs and PD

Parkinson’s disease (PD) is a common neurodegenerative disease characterized by low expression of dopamine in the striatum and degeneration of dopaminergic neurons in the substantia nigra pars compacta, leading to various motor and non-motor symptoms (Palm et al., 2012; Zhang et al., 2017). Inhibition or replacement of denatured neurons has always been considered to be an effective and standardized treatment (Palm et al., 2012). Although a variety of treatments are currently used to treat PD, such as levodopa therapy, deep brain stimulation surgery and rehabilitation, these treatments have been identified as current treatment strategies (Yasuhara et al., 2015; Yasuhara et al., 2017). Although these methods have certain therapeutic effects, they cannot prevent functional defects and disorders caused by dopamine depletion. Fortunately, cell therapy may be the key to the real recovery of degenerative/degenerative neurons. The cell therapy of PD began in 1979. The motor abnormality of PD rats was improved by transplanting dopamine-containing neurons from fetal rats. It was found that the transplanted cells survived and the axons grew well (Yasuhara et al., 2015). With the continuous study of cell therapy, researchers have transplanted different types of bioactive cells, such as olfactory sheath cells and NSCs, into the brain to produce therapeutic effects (Agrawal et al., 2004; Heris et al., 2022).

The number of neural progenitor cells/NSCs in the subependymal area, subgranular area and olfactory bulb of PD patients has been decreased after death, suggesting that the production of neural precursor cells/NSCs in patients with PD is impaired, which is the result of dopaminergic nerve loss (Höglinger et al., 2004; OSullivan et al., 2011). This suggests a close relationship between NSCs/neural progenitor cells and PD. Therefore, NSCs transplantation may be a new neural function recovery strategy for the treatment of PD. NSCs transplantation can be integrated into the surrounding environment of the central nervous system to establish an appropriate microenvironment to enhance dopaminergic nerve transmission (Chou et al., 2015). NSCs can differentiate into dopaminergic by recognizing the cellular and acellular regions of the target in the microenvironment. With the integration of differentiated dopaminergic neurons into the existing nigrostriatal dopaminergic pathway, the symptoms of PD can be alleviated (Chou et al., 2015). The NSCs/neural progenitor cells isolated from mesencephalic organs expanded stably and differentiated into mesencephalic dopamine neurons, showing better synaptic maturity and function. Further transplantation of NSCs/neural progenitor cells into the animal model of PD produced reproducible and effective therapeutic results (Kim et al., 2021). *In vitro* analysis showed that human NSCs line hVM1 clone 32 cells expressed standard dopamine-centered markers and other markers related to mitochondrial and peroxisome function and glucose and fat metabolism. NSCs were transplanted into PD mice to express dopamine-centered markers, the number of astrocytes and microglia increased significantly, and the weight of non-phosphorylated neurofilaments in motor cortex increased, which improved hyperactivity and gait changes in mice (Nelke et al., 2022). It is suggested that human NSCs transplantation has the function of preventing sports and non-sports injury. Moreover, in recent years, it has been found that the secretory group produced by NSCs plays a protective role in PD neurons. Injection of conditioned medium of NSCs into striatum and substantia nigra can improve motor and non-motor dysfunction in PD rats and save the loss of dopaminergic neurons (Ni et al., 2022). Conditioned medium of NSCs can reduce the apoptosis of PC12 cells induced by 6-OHDA, reduce the level of oxidative stress and improve the dysfunction of mitochondria (Ni et al., 2022). This may be related to the protective effect of conditioned medium of NSCs on 6-OHDA injured neurons through Sirt1 pathway.

Based on the role of NSCs in the treatment of PD. In order to optimize the survival of transplanted NSCs and guide their proper differentiation, researchers have combined different types of cells (such as endothelial cells and microglia) (Deng et al., 2013; Qian et al., 2020) and other factors (such as neurotrophin 3 (Gu et al., 2009)) with NSCs in the treatment of PD and achieved exciting results. Studies have shown that the combined transplantation of mesenchymal stem cells (MSCs) conditioned medium and NSCs can differentiate into dopaminergic neurons in the ventral tegmental area and medial forebrain bundle, migrate around the injured site, significantly reduce apomorphine-induced rotational asymmetry and improve spatial learning ability (Yao et al., 2016). Interestingly, combined transplantation showed higher activity in terms of cell survival, migration and behavior improvement than single NSCs transplantation (Yao et al., 2016). Other studies have shown that NSCs combined with ethyl stearate can promote the migration of NSCs from striatum to substantia nigra through CCL5/CCR5, and promote the differentiation of NSCs into dopaminergic neurons, thus improving the motor behavior of PD rats (Huang et al., 2023).

Although these studies reveal the potential value of NSCs in the treatment and application of PD, NSCs transplantation is still challenged. In the heterotopic even if the NSCs are transplanted into the primitive sites of PD, such as the substantia nigra and striatum, the new dopaminergic neurons are difficult to regenerate the axons of the striatum and are unlikely to integrate into the normal brain circuit, which leads to the main defects and obstacles of the current therapeutic effect. Another challenge is that NSCs therapy is how to target to promote the differentiation of transplanted NSCs into reliable and renewable dopamine neurons and to provide rich and effective cell substitutions for the treatment of PD.

### 4.2 NSCs and AD

Alzheimer’s disease (AD) can be described as a serious social, economic and medical crisis of our time. The core of the pathogenesis of AD lies in the following aspects. Tau is a microtubule-associated protein in neurons, which is very important for structural support and axonal transport. It is overphosphorylated, causing microtubules to collapse and gather into nerve fiber tangles. Secondly, β-amyloid (A-β) fragments accumulate and accumulate outside the cell. While activated microglia are closely related to β-amyloid aggregation and amyloid plaque formation. Thirdly, the loss of a large number of neurons and synapses is the most closely related factor of early cognitive decline in AD (Delbeuck et al., 2003; Salloway et al., 2014; Duncan and Valenzuela, 2017; Fang et al., 2019). AD is characterized by progressive neurodegeneration, mainly neuronal degeneration/degeneration and synaptic loss. Therefore, reversing and improving neuronal degeneration and axon loss are very important for the treatment of AD. With the emergence of cell therapy, it can not only prevent the development of the disease, but also reverse the symptoms, which has made some achievements in the application of AD.

The pathological changes of amyloid precursor protein in the progression of AD may prevent the neuronal differentiation of NSCs, while reducing the amyloid precursor protein can promote the neuronal differentiation of NSCs and migrate and differentiate into neurons in the hippocampus and cortex (Marutle et al., 2007). It also means that NSCs play an important role in AD process. NSCs transplantation plays an important therapeutic role in the application of AD by differentiating neurons, replacing and improving lost neurons. NSCs can enhance neuronal connectivity and metabolic activity in AD through compensation mechanism, thus saving the cognition of AD (Li et al., 2016). The neurospheres derived from mouse embryonic stem cells were transplanted into the frontal cortex of C57BL/6 mice. The results showed that the transplanted neurospheres survived and produced many ChAT positive neurons and a small number of serotonin positive neurons in and around the grafts (Wang et al., 2006). The working memory errors of mice were significantly reduced (Wang et al., 2006). This study revealed that the neurospheres derived from mouse embryonic stem cells significantly alleviated cholinergic deficit and short-term memory impairment in AD mice. NSCs are produced by growth factor-mediated selection in the absence of serum, and the induced NSCs can differentiate into cholinergic neurons (Moghadam et al., 2009). After transplantation of neural progenitor cells, the memory function of rats was significantly improved. Immunohistochemical analysis showed that most (about 70%) neural progenitor cells and/or primary neural progenitor cells retained neuronal phenotype after transplantation, of which about 40% had cholinergic phenotype and no tumor formation (Moghadam et al., 2009). It is suggested that NSCs transplantation is safe and effective. NSCs obtained from the hippocampus of rats were transplanted into the basal forebrain of AD rats. It was found that these cells could differentiate into neurons and astrocytes, and the number of cholinergic neurons increased, which improved the symptoms of AD rats (Xuan et al., 2008). It is interesting that further intracerebroventricular injection of brain-derived neurotrophic factor (BDNF) on the basis of NSCs transplantation can improve the differentiation of NSCs, improve the symptoms of AD rats, and improve the therapeutic effect of NSCs (Xuan et al., 2008). Further study also found that the transplanted NSCs from the hippocampus could differentiate into cholinergic neurons. NSCs transplantation could significantly increase the number of cholinergic neurons in the medial septal nucleus and vertical branch of diagonal branch, which improved the learning and memory ability of rats (Xuan et al., 2009).

NSCs transplantation can also inhibit neuroinflammation, cholinergic dysfunction, pericyte and synaptic loss, promote adult hippocampal neurogenesis and improve the cognitive impairment of AD (Lu et al., 2021). Human NSCs were repeatedly intranasally injected into the brain of APP/PS1 transgenic AD mice. The results showed that human NSCs transplanted into nasal cavity survived and showed extensive migration and high neuronal differentiation, while glial differentiation was relatively limited (Lu et al., 2021). NSCs transplantation can differentiate into cholinergic neurons, thus saving cholinergic dysfunction in APP/PS1 mice (Lu et al., 2021). In addition, nasal transplantation of human NSCs can improve other pathological changes similar to AD, including neuroinflammation, cholinergic dysfunction, pericyte and synaptic loss, promote adult hippocampal neurogenesis, and ultimately save the cognitive impairment of AD mice (Lu et al., 2021). The mechanism of NSCs transplantation to improve the symptoms of AD may reduce the accumulation of β-amyloid protein by up-regulating the expression of β-amyloid protease, insulin-degrading enzyme and Neprilysin. Transplantation of NSCs and bone marrow MSCs reduced the number of β-amyloid plaques in the hippocampus of AD mice and increased the polarization of M2 microglia, suggesting that stem cell transplantation has anti-inflammatory effects (Campos et al., 2022). Other studies have shown that NSCs transplantation reverses the overactivity in the hippocampus of AD mice, increases the number of microglia, and improves the learning and memory ability of mice (Campos et al., 2022). These studies highlight the potential benefits of NSCs in the treatment of AD.

### 4.3 NSCs and ALS

Amyotrophic lateral sclerosis (ALS) is a fatal disease characterized by persistent degeneration of motor neurons, which is characterized by paralysis and death by affecting motor neurons in the motor cortex, brainstem and spinal cord (Mazzini et al., 2016; Meyer, 2021). At present, some studies have proposed several pathological mechanisms of ALS-induced motoneuron death, including glutamate-induced excitotoxicity, abnormal cytoskeleton, protein aggregation, oxidative stress, mitochondrial dysfunction and extracellular SOD1 toxicity (Peters et al., 2015; Shafiq et al., 2021). There is no effective treatment for this devastating disease. With the exploration of the treatment of ALS, researchers have transplanted different types of functional cells to replace lost and degenerative neurons, resulting in therapeutic effects. In view of the unique characteristics of NSCs, which can differentiate into neurons, exciting results have been obtained in the therapeutic application of ALS (Kim et al., 2013; Baloh et al., 2018). NSCs transplanted into ALS animals can be preferentially colonized in motor cortex, hippocampus and spinal cord. Their differentiation is characterized by decreased expression of nestin, positive cells of MAP2, GFAP, O4 and CD68, and increased differentiation and survival of NSCs related to tumor necrosis factor (Mitrecić et al., 2010).

NSCs transplantation can slow down the onset and progression of ALS symptoms and prolong survival time. This beneficial effect seems to be achieved by producing nutritional factors, protecting neuromuscular function, reducing astrocyte proliferation, improving inflammation and enhancing synaptic plasticity (Teng et al., 2012). The *in vitro* characteristic of iPSCs-derived neural precursor cells is their ability to differentiate into neuronal phenotype. iPSCs-derived neural precursor cells transplantation normalized the expression of host genes (versican, has-1, tenascin-R, NGF, IGF-1, BDNF, bax, bcl2, and Casp-3), significantly preserved motor neurons, protected neural networks around motor neurons, slowed disease progression, and prolonged survival in all treated animals (Forostyak et al., 2020). The transplantation of human NSCs into the lumbar spinal cord of ALS rats can protect the α motoneurons near the transplanted cells and provide a transient functional improvement, but has no protective effect on the α motoneurons far away from the lumbar graft (Hefferan et al., 2012). Further studies showed that human NSCs was transplanted into the spinal cord and found that NSCs migrated and integrated widely in the spinal cord and differentiated into neurophenotypes, which delayed the loss of body weight and the deterioration of motor ability in ALS rats (Zalfa et al., 2019). NSCs transplantation reduced the number of activated astrocytes and microglia and increased the overall survival of rats. No rejection related to NSCs transplantation was observed (Zalfa et al., 2019). These studies have revealed the potential therapeutic effect of NSCs transplantation in the treatment of ALS, and it is a safe and feasible treatment.

However, people soon realized that this was an extremely difficult task, because newborn and differentiated motor neurons must project to distant axons, replace lost neurons and connect with host neurons, and reconstruct neural network function. Moreover, the toxic microenvironment formed in the process of ALS, including inflammatory attacks, has toxic effects on transplanted cells, resulting in reduced cell survival, which is also a difficult problem to be solved at present. Furthermore, how to control and enhance the targeted differentiation of transplanted NSCs into motor neuron phenotype, enhance synaptic plasticity and reduce their differentiation to other cell phenotypes is also a key issue in the treatment of ALS.

### 4.4 NSCs and HD

Huntington’s disease (HD) is a neurodegenerative disease caused by the CAG amplification of the Huntington protein (HTT) gene. The disease is characterized by neuronal degeneration in the striatum and cortex, leading to functional degeneration and disorders, including cognitive impairment and motor disorders (Bashir, 2019; Kim et al., 2021). With the development of the disease, the loss of neurons can affect many areas of the brain more widely (Kim et al., 2021). Since the discovery of HTT gene, no great progress has been made in the development of therapeutic strategies to prevent or slow down the progress of HD, and there is no effective treatment (Connor, 2018). With the biological mining of stem cells and their application in neurodegenerative diseases, some new and exciting results have emerged. With the biological mining of NSCs and their application in neurodegenerative diseases, some new and exciting results have emerged. The unique characteristics of NSCs provide the groundwork for the application and treatment of HD. The basis of NSCs therapy for HD is to replace or prevent cell loss. With the biological mining of NSCs and their application in neurological disease, some new and exciting results have emerged. The unique characteristics of NSCs provide the groundwork for the application and treatment of HD.

The role of NSCs transplantation in HD therapy is mainly through differentiation into neuronal phenotype, enhancement of neuronal plasticity and cell substitution (Li et al., 2020; Latoszek and Czeredys, 2021; Berlet et al., 2022). NSCs need to differentiate into gamma-aminobutyric acid (GABA) neuronal subtype before transplantation in the treatment of HD (Bosch et al., 2004). A population of homogenous functional GABA neurons was generated from neural stem cell lines. These neurons survive in HD and maintain their acquired fate (Bosch et al., 2004). In the model of HD, GABA neurons differentiated from NSCs can survive for a long time and make synaptic contact with endogenous neurons when transplanted into the striatum (Bosch et al., 2004). This reveals the possibility of using NSCs for cell replacement therapy for HD. Transplantation of NSCs derived from human embryonic stem cells into the striatum of HD model mice improved motor and cognitive impairment, saved synaptic changes, and connected with nerve endings of cells in the host (Reidling et al., 2018). Moreover, the implanted hNSCs had electrophysiological activity. HNSCs improved the motor and late cognitive impairment of HD model mice by reducing the abnormal accumulation of mutant HTT protein and the expression of brain-derived neurotrophic factor (BDNF) (Reidling et al., 2018). NSCs may reverse the progression of the disease by reducing the abnormal accumulation of mutant HTT protein and increasing the expression of brain-derived neurotrophic factor (BDNF) (Yoon et al., 2020). The immortalized NSCs could differentiate and express BDNF medium spiny neurons and GABA neurons in the striatum of HD animals, while the NSCs transplanted into the cortex formed Tbr1 positive neurons (Yoon et al., 2020). These transplanted NSCs can be stably implanted and connected in the host brain tissue, reducing the formation of glial scar and inflammation, and increasing endogenous neurogenesis and angiogenesis (Yoon et al., 2020). This study suggests that a clinical grade NSCs line is effective in preclinical trials of HD. These studies have revealed the safety and effectiveness of NSCs transplantation in HD.

Autologous or allogeneic NSCs are limited and ethically limited, while iPSCs to derive/differentiate NSCs not only provides a rich cellular guarantee in source, but also suppresses ethical problems. Indeed, NSCs derived from iPSCs have also made some achievements in HD therapy (Liu et al., 2017; Cho et al., 2019). iPSCs were induced to differentiate into NSCs *in vitro*. After transplantation into HD mice, it was found that these cells could survive *in vivo* and differentiate into region-specific neurons (moderately prickly neurons), which improved the motor dysfunction of mice (Al-Gharaibeh et al., 2017). Other studies have also found that neural progenitor cells derived from human leukocyte antigen homozygote-iPSCs are transplanted into the striatum of HD mice. It is found that the transplanted cells have multiple differentiation potential (differentiation into neurons, oligodendrocytes and astrocytes) (Park et al., 2021). These cells restored the expression of DARPP-32, the density of synaptophysin, the expression of myelin basic protein in corpus callosum and the function of astrocytes, and played a role in neuroprotection and functional recovery (Park et al., 2021). This suggests that NSCs derived from iPSCs may provide a promising therapeutic strategy for neuronal replacement of HD.

Although encouraging achievements have been made in the application of NSCs in HD therapy, the wide application and development of cell replacement therapy is limited due to some inherent problems of cells (such as genetic instability and safety of NSCs derived from iPSCs and autologous cell source) and the technical differences in the use of NSCs in laboratory. Nevertheless, NSCs are a new cell substitution strategy and have a wide range of development and application value in the application of HD therapy.

### 4.5 NSCs and peripheral nerve injury (PNI)

The process of regeneration and repair of PNI is a dynamic process regulated by multi-cell and multi-molecule, involving neurons, SCs (repair phenotype) and immune cells (macrophage activation and phagocytosis). The interaction between these cells regulates the process of peripheral nerve regeneration and repair (Chen et al., 2015; Modrak et al., 2020; Nocera and Jacob, 2020). In this process, neuronal degeneration, axonal atrophy and demyelination are the key factors leading to the obstruction of PNI regeneration, and other factors include inflammatory reaction, glial scar formation and immunity, which can hinder peripheral nerve regeneration and repair (Modrak et al., 2020; Zhang et al., 2023). It is understood that the inherent ability of PNI regeneration is limited, lack of regeneration basis and environment. Therefore, it is very important to improve and provide the microenvironment for PNI regeneration and promote axonal regeneration and myelination. NSCs have shown their ability to promote nerve regeneration and repair tissue injury in most studies. The mechanisms of NSCs promoting nerve regeneration include neurotrophic factors secreted by NSCs (such as BDNF, NGF, neurotrophin 3/4 and vascular growth factor), anti-inflammatory and immunomodulatory, axonal regeneration and remyelination, neuroprotection and improving the local microenvironment of nerve injury, which are beneficial to the regeneration and repair of PNI (Sullivan et al., 2016; Assinck et al., 2017; Pisciotta et al., 2020; Zhang et al., 2020).

NSCs transplantation can significantly improve the formation of axonal myelin in sciatic nerve injury and regeneration, and increase the expression of hepatocyte growth factor (HGF) and nerve growth factor (NGF) (Cheng et al., 2011; Xu et al., 2012). Extraction, isolation and identification of functional NSCs from human iPSCs injected into denervated gastrocnemius muscle enhanced neuromuscular regeneration after denervation (Li et al., 2022).

The dry weight and muscle fiber area of gastrocnemius muscle increased significantly at 3, 5 and 7 months after NSCs transplantation. 7 months after embryonic NSCs transplantation, neurons can still be seen in the distal tibial nerve (Gu et al., 2010). It is suggested that embryonic NSCs can differentiate into neurons and form functional neuromuscular connections with denervated muscles, which may be beneficial to the treatment of muscle atrophy after peripheral nerve injury (Gu et al., 2010). All these show that NSCs have obvious advantages and therapeutic potential in promoting nerve regeneration after PNI. Interestingly, different passages of NSCs are different in the treatment of PNI. Human NSCs (fifth or sixth generation) were transplanted into 15 mm rat sciatic nerve injury model to compare the effects of two passage cell lines in the treatment of nerve injury. Compared with the fifth generation, the survival rate, SCs differentiation, axonal growth and functional results of the sixth generation of transplanted cells decreased (Du et al., 2018). The therapeutic effect of the sixth generation of NSCs on peripheral nerve regeneration was significantly lower than that of the fifth generation (Du et al., 2018). This study means that there are differences in PNI regeneration and repair among different passages of NSCs, which may be related to the decrease of cell viability and biological activity caused by long passage time of cell culture.

In view of the functional role of NSCs in the repair of PNI. Some studies explore the efficacy of NSCs in the treatment of PNI by combining other cells (such as olfactory ensheathing cells) or other factors/substances (such as overexpression of glial-derived neurotrophic factor, catheter/scaffold and matrix). These combined transplantation can enhance the therapeutic effect of NSCs and exert the characteristics of synergistic treatment (Shi et al., 2009; Syu et al., 2019; Zhang et al., 2019). *In vitro*, Estradiol promotes the migration and proliferation of NSC and inhibits apoptosis (Sekiguchi et al., 2013). The combined application of Estradiol and NSCs transplantation can promote the recovery of injured peripheral nerve function, increase the amount of vascular and blood perfusion in the nerve, and accelerate the motor nerve conduction velocity, voltage amplitude and exercise tolerance (Sekiguchi et al., 2013). Laminin-chitosan-PLGA nerve conduit containing SCs and NSCs can promote the regeneration of recurrent laryngeal nerve injury, and the effect is better than that of autogenous nerve transplantation (Li et al., 2018; Li et al., 2020). The diameter and area of regenerated myelin sheath of recurrent laryngeal nerve and the secretion of brain-derived neurotrophic factor and glial cell line-derived neurotrophic factor in laryngeal muscle or regenerated nerve tissue were significantly increased after transplantation of laminin-chitosan-PLGA nerve conduit containing Schwann cells and NSCs (Li et al., 2020). Co-culture of Schwann cells and NSCs in laminin-chitosan-PLGA catheter may promote the regeneration of injured nerves (Li et al., 2020). This method may be a promising alternative to repair defective nerves. Co-inoculation of NSCs and SCs into PLGA catheter carriers carrying NT-3 promoted NSCs to differentiate into neurons, enhanced synaptic connections and synaptic activity, and accompanied by SCs myelinated neurite processes (Xiong et al., 2012). This may be due to the fact that the catheter/stent can support and guide the growth of regenerated axons from one nerve stump to another, while preventing the growth of fibrous tissue and retaining neurotrophic factor components.

It is understood that SCs constitute the myelin cells of the peripheral nerve and play an important role in protecting neuronal axons and vegetative neurons. SCs play an irreplaceable role in the regeneration and repair of peripheral nerve. In addition, some researchers can promote axonal regeneration and nerve repair by transplanting SCs into PNI (Salehi et al., 2018; Yu et al., 2022; Chen et al., 2023). The differentiated NSCs showed spindle-like morphology similar to SCs and expressed glial cell markers p75 and S100 (Tong et al., 2010). SCs differentiated from NSCs can promote axonal growth (Tong et al., 2010). This suggests that NSCs can differentiate into SCs with similar morphology, phenotype and function. Interestingly, the implanted NSCs can differentiate into SCs and maintain a microenvironment rich in growth factors, thus promoting nerve regeneration (Lee et al., 2017). *In vitro* experiments showed that IL12p80 (homodimer of IL12p40) promoted the differentiation of mouse NSCs into SCs through signal transduction and phosphorylation of activator of transcription 3 (STAT3) (Lee et al., 2017). In the mouse model of sciatic nerve injury, the combined transplantation of NSCs with neural conduit and IL12p80 could promote motor recovery and increase the medial diameter of regenerated nerve by 4.5 times (Lee et al., 2017). Other studies have also revealed that co-culture of SCs and NSCs can promote NSCs to differentiate into neurons and secrete higher levels of neurotrophic factors including BDNF and GDNF (Yu et al., 2017). All these reveal the fact that NSCs can not only differentiate into other cell phenotypes, but also differentiate into SCs to promote and participate in the repair of PNI. The above studies show that NSCs can be used as ideal candidate cells for PNI regeneration and have a great application prospect in PNI regeneration and repair.

## 5 Clinical application and challenges of NSCs in neurological diseases

The treatment of neurological diseases has always been a thorny problem in clinic, and it is very important to stop and delay the progress of these diseases. Unfortunately, although drugs or other methods are often used in clinic to alleviate the progression of the disease, the therapeutic effect is still not satisfactory, which brings a great challenge to the clinic. It is important is that these methods cannot replace the lost/degenerated neurons, restore the stability of the internal environment and reconstruct the neural network, so there are great defects in the treatment. In addition, there are inherent defects in long-term drug use, such as dependence, side effects and cut-off phenomena, and huge economic burden, which lead to slow progress in clinical treatment. The emergence of cell replacement therapy has opened up a bright future for the treatment of neurological diseases. The unique biological characteristics of NSCs and their great potential in the treatment of neurological diseases have been favored by many researchers. As mentioned earlier, in different basic research fields, NSCs transplantation has a therapeutic effect on neurological diseases, which can reverse and improve disease progression and repair function. In view of the substantial results of basic research, researchers have risen from basic research to the exploration stage of clinical trials to further explore the effectiveness, safety and feasibility of NSCs in clinical trials.

Although the reports of NSCs in clinical trials of neurological diseases are limited, but some clinical trials have reported the potential value of NSCs in the treatment of neurological diseases and achieved some encouraging results (Glass et al., 2012; Luan et al., 2012; Riley et al., 2012). In a phase 1 clinical trial, 12 patients with ALS received intraspinal injection of NSCs. After a follow-up of 30 months, these patients received MRI assessment, standard clinical evaluation and regular functional evaluation, including ALSFRS-R, sitting forced vital capacity, grip strength assessment, hand dynamometer, electrical impedance myogram and bladder ultrasound. The results showed good tolerance and the feasibility and safety of cell transplantation, which delayed the progression of the disease (Feldman et al., 2014). A clinical trial in which T cell vaccines combined with autologous NSCs were transplanted into 7 patients with ALS. The patients were evaluated by using rating scale (ALSFRS-R) and neurological function. Results showed that cell transplantation could slow down or stop the progression of the disease. The median survival time of the patients was prolonged from 3.5 years to 6 years, and the improvement of neurological function lasted for at least 1 year. It is considered that cell transplantation may be a feasible, minimally invasive, safe and effective method, and can obtain lasting curative effect (Moviglia et al., 2012). Another clinical trial found that fetal NSCs were transplanted into the spinal cord of 6 patients with ALS. The main outcome measure is short-term and long-term security. mortality and severe adverse reactions of any cause, while secondary outcome measures were ALS-FRS-R scale and forced vital capacity (FVC) measurement. No increase in disease progression was found during the 18-month follow-up after transplantation (Mazzini et al., 2015). The ALS-FRS-R scale of two patients (from 1 to 2) showed a transient improvement in walking. One patient’s tibialis anterior muscle MRC score improved for 7 months (Mazzini et al., 2015). Two patients refused to accept PEG and invasive ventilator and died of respiratory failure 8 months after operation, confirming that the death was related to the progression of the disease (Mazzini et al., 2015). It is suggested that NSCs transplantation is a safe method of cell therapy. 18 patients with ALS received human NSCs transplantation. Patients before and after transplantation were monitored by clinical, psychological, neuroradiological and neurophysiological evaluation. None of the patients showed serious adverse reactions or accelerated disease progression during the 60-month follow-up (Mazzini et al., 2019). It also reflects that transplanting NSCs is a safe method and will not have significant harmful effects in the short and long term. However, other clinical trials have found that NSCs transplantation can bring related side effects. In the phase 1/2 clinical trial, it is safe and feasible that NSCs can be transplanted into 15 patients with ALS through spinal canal at high dose. The patients were followed up for up to 24 months and assessed for adverse events and disease progression, measured by the ALS functional rating scale, a revised quantitative measurement of forced vital capacity and strength (Glass et al., 2016). However, one patient has transient pain and side effects of immunosuppressive, one patient has acute neurological deterioration and the other has central pain syndrome (Glass et al., 2016).

A clinical trial reported the effect of NSCs transplantation in a patient with PD. Human neural progenitor cells/undifferentiated NSCs were transplanted into the dorsal putamen of 8 patients with moderate PD. The patients were evaluated by neurology, neuropsychology and brain imaging before operation and 1 year, 2 years and 4 years after operation. It was proved that the cell transplantation was safe, there was no immune reaction to the transplantation, and there was no adverse reaction (Madrazo et al., 2019). The patient’s neuropsychological score was not affected by the transplantation (Madrazo et al., 2019). The motor function of all but one of the patients was improved in varying degrees, and the response of five patients to L-dopa showed better curative effect (Madrazo et al., 2019). PET imaging showed a tendency to increase dopaminergic activity in the midbrain after cell transplantation (Madrazo et al., 2019). Other clinical trials reported that NSCs were transplanted into the striatum of 21 patients with PD. Unified Parkinson’s Disease Rating Scale (UPDRS), Hoehn-Yahr, PDQ-39 and Schwab-England Scores were used to evaluate the Parkinson patients’ neurofunctions. The possible side effects of transplantation were evaluated from four aspects: a) tumor formation, b) immune rejection and the use of immunosuppressants, c) graft complications and d) side effects related to delivery. The symptoms of all PD patients were significantly improved, and there were no obvious side effects including tumorigenesis, immune rejection and complications after transplantation (Lige and Zengmin, 2016) (Table 3).

**TABLE 3:** Clinical trial of NSCs in neurological diseases.

NSCs source	Disease type	Transplant method and site	Transplant dose	Number of patients	Follow-up time	Evaluation methods and therapeutic effect	Side effect	Refs
Fetal NSCs	ALS patients	Lumbar intraspinal injection	100,000 cells at a time (5 times unilateral or bilateral)	12	6–18 months	Patients passed standard clinical examination and quarterly evaluation, including ALSFRS-R, HHD, FVC, EIM and bladder ultrasound examination for residual urination. MRI scanning of surgical area. There is no evidence that neural stem cell transplantation accelerates the progression of the disease. The clinical condition of one patient improved	Some patients experienced transient or transient nerve root pain and/or sensory abnormalities and were relieved at discharge	Glass et al. (2012)
NSCs derived from fetal spinal cord	ALS patients	Lumbar intraspinal injection	5 injections (10 μL/time), each injection containing 100,000 cells	12	8–13 months	Patients were followed up clinically and radiologically to assess the potential toxicity of the operation. Microinjection of NSCs into unilateral and bilateral lumbar spinal canal is safe and feasible. No recorded motor dysfunction was found at discharge	Transient somatosensory evoked potential inhibition occurred in 1 case. One case of urinary retention required reinsertion of Foley catheter	Riley et al. (2012)
NSI-566RSC human NSCs	ALS patients	5 unilateral cervical injections or 10 bilateral lumbar injections	100,000 NSCs were injected at a time, with a dose range of up to 1.5 million cells	12	6–15 months	Patients were measured by detailed neurological results before and after operation. NSCs transplantation will not accelerate the disease progression of the subjects, which verifies the safety and feasibility of cervical and lumbar injection	There are no related reports	Feldman et al. (2014)
GMP-grade fetal NSCs	ALS patients	3 unilateral or 3 bilateral intrafascicular injections	50,000 cells/μL	6	18 months	Patients were evaluated by using the ALS-FRS-R scale. The ALS-FRS-R scale of two patients (from 1 to 2) showed a transient improvement in walking. The tibialis anterior muscle MRC score of the third patient improved for 7 months. The other two patients refused to accept polyethylene glycol and invasive ventilator and died of respiratory failure 8 months after operation. The autopsy confirmed that this was related to the evolution of the disease	No one showed serious adverse reactions related to treatment	Mazzini et al. (2015)
NSCsderived from neurospheres	ALS patients	Microinjection into the gray matter bundle of lumbar or cervical spinal cord	50,000 cells	21	60 months	Patients before and after transplantation were monitored by clinical, psychological, neuroradiological and neurophysiological evaluation. Transplanting NSCs is a safe method and will not have significant harmful effects in the short and long term	None of the patients showed serious adverse reactions or accelerated disease progression due to treatment	Mazzini et al. (2019)
Fetal NSCs	ALS patients	Bilateral cervical spinal cord/lumbar vertebra injection	100,000 cells (10ul)	15	3–24 months	Patients were assessed for adverse events and disease progression, measured by the ALS functional rating scale, a revised quantitative measurement of forced vital capacity and strength. Intraspinal transplantation of NSCs can be safely completed at high dose	Transient pain is associated with the side effects of immunosuppressive drugs. One case of acute neurological deterioration and another case of central pain syndrome	Glass et al. (2016)
Human neural precursor cells derived from human fetal brain tissue	PD patients	Bilateral intradural transplantation	2×10^6^ cells (2 mL)	8	4 years	The patients were evaluated by neurology, neuropsychology and brain imaging. Exercise of all patients was improved in varying degrees, and five of them responded better to the drugs. PET indicates that dopaminergic neurotransmission in putamen is enhanced, which may be related to the improvement of motor function and has a better response to L dopamine	No adverse reactions were found	Madrazo et al. (2019)
Differentiation of embryonic stem cells	PD patients	Unilateral striatal implantation	3×10^7^ cells, per unit of 0.25 mL	21	57 months	Unified Parkinson’s Disease Rating Scale (UPDRS), Hoehn-Yahr, PDQ-39 and Schwab-England Scores were used to evaluate the Parkinson patients’ neurofunctions. The symptoms of PD patients were significantly improved after NSCs transplantation, and it is considered that NSCs transplantation may be a safe and effective method	There are no obvious side effects of transplantation	Lige and Zengmin (2016)
NSCs line NSI-566RSC	ALS patients	Injection of ventral horn of cervical and thoracolumbar spinal cord	100,000 cells (10ul)	15	13 months	All patients who received intraspinal cord transplantation were well tolerated	One patient developed kyphosis and underwent laminoplasty. Another case required reoperation because of wound dehiscence and infection. 1 patient needs to be intubated again	Riley et al. (2014)

These clinical trials reflect the feasibility of NSCs transplantation in clinical trials of neurological diseases, but the safety and efficacy of NSCs transplantation are still not very clear, and there is a lack of more direct clinical data to support it. Although NSCs have found great potential therapeutic value in animal basic experiments and obtained encouraging favorable results, they ignore the differences and diversity of humans. It is not clear whether the positive and beneficial results obtained in animal studies will achieve similar results in human clinical trials, and there is a lack of more clinical data. Otherwise, it is not feasible to introduce it directly into the human body for granted. At this stage, NSCs are not widely used in clinical trials, which may be related to some problems existing in stem cell transplantation and hinder the development of clinical trials.

First, a rich and effective source of cells is the primary problem that limits the wide range of clinical development. The natural limitations of cell source and *in vitro* expansion are still the main challenges to the development of autologous cell transplantation. The sources of autologous or allogeneic fetal NSCs are limited and ethically limited. Although NSCs can be derived from reprogramming or iPSCs, appropriate manufacturing processes must be established and certified to ensure that they range from research to therapeutic development and application. However, the lack of consensus on the characteristics of these neural stem cells and the methods used to evaluate them is still a controversial issue. Moreover, the transplantation of these derived NSCs into the body has certain safety risks and side effects, including tumor formation, genetic instability and related complications after transplantation. Therefore, for cells from these sources, cell characteristics and genetic fidelity should be evaluated to avoid cell line switching, cross contamination and carrying chromosome rearrangement. Karyotype analysis and short tandem repeat analysis are the simplest techniques to achieve this purpose. therefore, it should be carried out routinely.

In addition, the cell culture cycle *in vitro* is relatively long, the culture system is still imperfect and lack of efficiency, which may miss the key treatment window []. Therefore, *in vitro* and purified cells need relatively short expansion culture, enhance the viability and proliferation of cultured cells, improve the culture system *in vitro*, and obtain abundant cells more efficiently. The formulation of strict culture programs and strict quality control may help us to achieve this goal. Moreover, studies have shown that there are differences in the treatment of neurological diseases in different passage lines of NSCs culture (Du et al., 2018). However, it is not clear which generation of cultured cells can achieve the best therapeutic effect, which needs to be explored and completed.

Second, the method of cell transplantation and the problem of location and tracking *in vivo*. NSCs can be transplanted into the body through a variety of methods and techniques, including intracerebral/spinal cord direct injection, intravenous transplantation, subarachnoid space transplantation and local injury site direct transplantation (Zhang et al., 2023). Although these methods can be well implemented by surgeons, there are differences in the therapeutic effects of different transplantation methods. For example, cells from vein transplantation or subarachnoid space transplantation can flow to other parts through the vein, and few of them may be colonized in the nerve injury site to play a therapeutic role. Currently, it is uncertain which transplantation method has the best therapeutic effect, and there is a lack of consensus. In addition, the transplanted cells cannot be tracked *in vivo*, and it is impossible to determine whether the transplanted cells are accurately colonized at the injured site, which also leads to a problem in the application of cell transplantation at present.

Third, the stability and safety of NSCs transplantation. Studies have shown that transplantation of NSCs into patients can lead to certain side effects, such as transient pain, central pain syndrome, immunosuppression and acute neurological deterioration (Riley et al., 2014; Glass et al., 2016). Therefore, the wide application of NSCs in clinical trials requires more animal research, fully understand the biological characteristics and safety of NSCs *in vivo*, and reduce the risk of additional injury. Thus, a good understanding of how NSCs work *in vivo* and how they connect and integrate with host cells, overcome tissue and environment-specific barriers, better characterize and control NSCs differentiation *in vivo*, target cell subtypes (neuronal subtypes) required for differentiation, and interact with the host.

Moreover, the time and dose of cell transplantation can lead to some differences or poor therapeutic effects. At present, there is a lack of guidance and consensus on the application of transplantation time and dose, which also needs more experimental work to explore in the future. Although the replacement therapy of NSCs transplantation is in the primary stage of exploration, there are many uncertain factors and many problems that need to be solved, but these will not prevent researchers from carrying out in-depth exploration and intensified work. in the end, these problems will be well solved.

## 6 Conclusion

In view of the differentiation potential of NSCs and their unique characteristics in the regeneration and repair of nerve injury, NSCs have great potential in the treatment of neurological diseases. NSCs transplantation has made great progress in the treatment of neurological diseases, revealing the fact that they can be a candidate cell replacement therapy for neurological diseases. However, NSCs are not ready for clinical transplantation. Although a few clinical trials have reported that NSCs are feasible and safe in the treatment of patients with neurological diseases, their wide application is still greatly hindered, and more direct evidence is needed to support it. Although there are many problems that need to be solved at present, they will not hinder the pace of researchers’ exploration, and eventually these problems will be well solved. In a word, neural stem cells are a promising cell transplantation therapy for the treatment of neurological diseases.
